# Impact of different biologically-adapted radiotherapy strategies on tumor control evaluated with a tumor response model

**DOI:** 10.1371/journal.pone.0196310

**Published:** 2018-04-26

**Authors:** Araceli Gago-Arias, Beatriz Sánchez-Nieto, Ignacio Espinoza, Christian P. Karger, Juan Pardo-Montero

**Affiliations:** 1 Instituto de Física, Pontificia Universidad Católica de Chile, Santiago, Chile; 2 National Center for Radiation Research in Oncology (NCRO), Heidelberg Institute for Radiation Oncology (HIRO), Heidelberg, Germany; 3 Grupo de Imaxe Molecular, Instituto de Investigación Sanitaria (IDIS), Santiago de Compostela, Spain; 4 Servizo de Radiofísica e Protección Radiolóxica, Complexo Hospitalario Universitario de Santiago de Compostela, Santiago de Compostela, Spain; North Shore Long Island Jewish Health System, UNITED STATES

## Abstract

Motivated by the capabilities of modern radiotherapy techniques and by the recent developments of functional imaging techniques, dose painting by numbers (DPBN) was proposed to treat tumors with heterogeneous biological characteristics. This work studies different DPBN optimization techniques for virtual head and neck tumors assessing tumor response in terms of cell survival and tumor control probability with a previously published tumor response model (TRM). Uniform doses of 2 Gy are redistributed according to the microscopic oxygen distribution and the density distribution of tumor cells in four virtual tumors with different biological characteristics. In addition, two different optimization objective functions are investigated, which: i) minimize tumor cell survival (OF_surv_) or; ii) maximize the homogeneity of the density of surviving tumor cells (OF_std_). Several adaptive schemes, ranging from single to daily dose optimization, are studied and the treatment response is compared to that of the uniform dose. The results show that the benefit of DPBN treatments depends on the tumor reoxygenation capability, which strongly differed among the set of virtual tumors investigated. The difference between daily (fraction by fraction) and three weekly optimizations (at the beginning of weeks 1, 3 and 4) was found to be small, and higher benefit was observed for the treatments optimized using OF_surv_. This in silico study corroborates the hypothesis that DPBN may be beneficial for treatments of tumors which show reoxygenation during treatment, and that a few optimizations may be sufficient to achieve this therapeutic benefit.

## Introduction

During the last years, several studies have provided clinical evidence for the non-uniform response of tumors to radiation, mainly caused by tumor-specific heterogeneity factors such as varying oxygen supply and tumor cell proliferation and density [1]. Among these, tumor oxygenation is important because of the increased radioresistance of hypoxic cells, which can seriously affect radiotherapy treatment outcome [2–4]. In these cases, dose escalation would be required to maintain the same level of tumor control probability as compared to well-oxygenated tumors. This may, however, produce unacceptable levels of normal tissue toxicity [5, 6]. A similar situation may occur in tumors presenting a non-uniform tumor cell density distribution. As an alternative to this homogeneous dose escalation, the delivery of non-uniform dose distributions was proposed long ago to increase the efficiency of radiotherapy [7–9]. Nowadays this approach is becoming more realistic due to the capabilities of modern delivery techniques, such as IMRT. The effect associated to the delivery of non-uniform dose distributions has been evaluated both in terms of TCP and BED [10, 11]. Moreover, functional imaging techniques providing spatial-temporal information associated to biological tumor properties can provide input data for biological optimization strategies [12–14].

Despite the experimental difficulties, several studies have shown the technical feasibility to prescribe and deliver inhomogeneous dose distributions based on functional imaging (see for example the review on dose painting by Shi *et al.* [15]). Among others, dose painting by contours (DPBC) and dose painting by numbers (DPBN) are terms that are most commonly used to refer to these techniques. The DPBC technique identifies one or several regions of the planned target volume with a higher probability of local recurrence. These subvolumes are then defined as subtargets to be treated with an additional uniformly distributed dose boost [16, 17]. DPBN on the other hand, involves a voxel-based dose prescription that is calculated based on the biological information provided by functional imaging [18, 19]. Two different DPBN-strategies have been proposed: i) delivering additional dose to the less radiosensitive cells [20–22] or; ii) redistributing the dose while keeping the integral dose to the target volume constant [23, 24].

For the implementation of either DPBC and DPBN, several methodologies have been proposed to guide dose prescriptions based on functional imaging, considering: i) tumor metabolism [21, 25–29], ii) oxygenation status [16, 30–32], and iii) proliferation of tumor cells [33, 34]. Usually, they parameterize tumor control probability (TCP) or design a dose prescription function based on tracer uptakes [22, 29, 35–38].

Comparing results from the works that have addressed the clinical implementation of dose painting is however a very difficult task. Many works involved dose escalation to different dose levels, applying different adaptive schemes, and aiming at different endpoints: locoregional tumor control improvement [27, 39, 40], maximun tolerated dose [25, 41], normal tissue toxicity reduction preserving tumor control [28, 42–44], palliative response [45], etc. For this reason, and in spite of the intense research dedicated to dose painting during the last years, procedures are far from being standardized. Under such scenario, radiobiological modeling is a powerful tool that can help to study different aspects of dose painting like adaptive scheme, tumor microenvironment changes, dose prescription algorithms, etc.

In this modeling study we investigate different DPBN strategies targeting hypoxic tumors. To simulate tumor response to uniform and non-uniform dose distributions we use a previously published computational model that considers several biological processes [46]. The main objective of the study is to determine the treatment gain that could be achieved with different number of treatment adaptations (optimization). As the clinical implementation of dose painting treatments involving a large number of optimizations is non realistic nowadays due to the high logistic effort (both in terms of human resources and functional imaging tests) finding a realistic compromise between treatment gain and number of biological optimizations is mandatory.

Other relevant aspect is the method used for dose distribution optimization. In general, the spatial dose distribution in the target is a result of an optimization problem governed by an objective function (OF). Two of the most frequently applied radiobiological optimization strategies are: i) to minimize the overall tumor cell survival (in this work referred as OF_surv_) [9, 20, 24] and; ii) to search for the dose distribution leading to a uniform tumor cell density (OF_std_). The latter approach assumes that a dose distribution designed to reduce biological heterogeneity within the tumor maximizes the TCP [23, 37, 47]. Although both optimization strategies would lead to a TCP higher than that of a uniform dose, the gain in treatment outcome for the different OF has not been investigated under a common methodology.

## Materials and methods

Dose painting optimization methods generally involve models of cell survival, which are based on the linear quadratic (LQ) model [48]. This model accounts for the oxygen effect through oxygen enhancement ratios (OER), which are modulating factors of the fully oxic radiosensitivity parameters *α* and *β*. Using the expression of Wouters and Brown for the OERs [49], the expression for the cell survival fraction (SF) can be written as follows:

$$\begin{array}{c} SF=exp[-\alpha_{h}OER_{\alpha }\left(p\right)d-\beta_{h}{\left(OER_{\beta }\left(p\right)d\right)}^{2}] \end{array}$$ (1)

where *d* is the fractional dose, *p* is the oxygen partial tension (*pO*_2_), *α*_*h*_ and *β*_*h*_ are the radiosensitivity parameters under hypoxic conditions and OER_*α*_ and *OER*_*β*_ are the oxygen enhancement ratios for *α* and *β*, respectively:

$$\begin{array}{c} OER_{\alpha (\beta )}=\frac{k+p\cdot OER_{\alpha_{m}\left(\beta_{m}\right)}}{p+k} \end{array}$$ (2)

Here, $OER_{\alpha_{m}}$ and $OER_{\beta_{m}}$ are the maximum values of *OER*_*α*_ and *OER*_*β*_ under aerobic conditions and *k* is the parameter determining the slope of the curve, *i.e.* the change of OER with *p*. In Eq (1), *α*_h_ and *β*_h_ are equal to $\alpha /OER_{\alpha_{m}}$ and $\beta /{\left(OER_{\beta_{m}}\right)}^{2}$ respectively.

Regarding the oxygen tension in the tumor, the microscopic oxygen distribution rather than the average *pO*_2_ value in a voxel should be used to calculate the oxygen-dependent tumor response [24]. The reason for this is that *pO*_2_ values may vary on a scale much smaller than the typical voxel size and the tumor response will be governed by the most hypoxic and thus most radioresistant cells. In this work, oxygen distributions are generated by a previously published Tumor Oxygenation Model (TOM) [50] depending on the vascular fraction (vf), which is defined as tumor vascular volume fraction in the voxel, and the oxygen consumption rate. To allow efficient calculations, oxygen distributions, condensed into 16 bin *pO*_2_-histograms, are assigned to each tumor voxel (see below).

For a virtual tumor consisting of *N* voxels with *j* levels of *pO*_2_ each, the cell survival *C*_surv_ is quantified as,

$$\begin{array}{c} C_{\text{surv}}=\sum_{i=1}^{N}\sum_{j=1}^{16}c_{i,j}\cdot exp\left[-\alpha_{h}OER_{\alpha }\left(p_{k}\right)d_{i}-\beta_{h}{\left(OER_{\beta }\left(p_{j}\right)d_{i}\right)}^{2}\right] \end{array}$$ (3)

where *c*_*i,j*_ is the number of tumor cells in voxel *i* with an oxygen pressure *p*_*j*_ corresponding to the *j-th* bin of the *pO*_2_-histogram assigned to the voxel, and *d*_i_ is the dose delivered to the voxel *i*.

### Optimization problem

Considering the cell survival presented in Eq (3), two dose redistribution optimization approaches were implemented using two different objective functions (OFs). Both are subject to the following dose constraints:

1. The average (integral) dose delivered to the virtual tumor is kept constant and equal to 2 Gy per fraction. The choice of dose redistribution, rather than dose escalation, allows comparing the results with that of standard fractionated treatments. Considering a boost would produce a higher effectiveness, not only because of the dose distribution modulation but also because of the increased energy deposition, *i.e.* integral dose to the tumor [23].
2. Dose modulation within the tumor: maximum and minimum doses (*d*_max_ and *d*_min_) are limited to differ by ±25% from the prescribed dose (2 Gy).

The objective functions investigated involved the following aproaches:

1. Minimizing the number of surviving cells (OF_surv_):
  The dose distributions minimizing the cell survival were determined according to,
  $$\begin{array}{c} d_{i}=\underset{d_{\text{min}}\leq d_{i}\leq d_{\text{max}}}{\text{argmin}}C_{\text{surv}},\;\;\text{subject}\;\text{to}\;\;d=\frac{1}{N}\sum_{i=1}^{N}d_{i}=2Gy \end{array}$$ (4)
2. Minimizing heterogeneity of surviving cells (OF_std_):
  The dose distributions leading to the highest spatial uniformity of cell survival were calculated by minimizing the standard deviation (*std*) of the number of surviving tumor cells within the tumor as,
  $$\begin{array}{c} d_{i}=\underset{d_{\text{min}}\leq d_{i}\leq d_{\text{max}}}{\text{argmin}}std\left(C_{\text{surv}}\right),\;\;\text{subject}\;\text{to}\;\;d=\frac{1}{N}\sum_{i=1}^{N}d_{i}=2Gy \end{array}$$ (5)

The non-linear constrained optimization problems defined by Eqs (4) and (5) were solved using the FILTER Sequential nonlinearly constrained optimization algorithm available on the NEOS server [51, 52].

### Tumor model (TOM and TMR)

In order to test the dose painting optimization strategies described above, a virtual head and neck (H&N) tumor was generated using the previously published Tumor Oxygenation Model (TOM) [50] and the Tumor Response Model (TRM) [46]. These simulation programs were developed to describe the spatial-temporal development of a given tumor, based on its biological parameters, but it may also be used to generate a virtual tumor with specified biological properties. A brief description of TOM and TRM is given in the following sections.

#### TOM

In this tool, microscopic *pO*_2_ distributions are calculated in a reference tumor volume (voxel) by solving a reaction-diffusion equation [50]. This voxel is assumed to contain parallely aligned and randomly distributed linear vessels as sources of oxygen and oxygen-consuming cells outside the vessels. *pO*_2_ distributions are calculated considering the vascular fraction, the intravascular *pO*_2_ and the oxygen consumption rate, which depends on the fraction of dead (*i.e.*, non-consuming) cells. Oxygen distributions, summarized in *pO*_2_-histograms, are pre-calculated for a set of vascular and dead cells fractions and are stored for further use by the TRM.

#### TRM

This voxel-based multiscale model simulates the growth and radiation response of hypoxic tumors [46]. It considers viable and dead tumor cells, capillary and normal cells, as well as the most relevant biological processes such as: i) proliferation of tumor cells; ii) hypoxia-induced angiogenesis; iii) spatial exchange of cells between neighbouring voxels leading to tumor growth; iv) oxygen-dependent radiation response according to Eq (1); v) resorption of dead cells and; vi) spatial exchange of cells between neighbouring voxels leading to tumor shrinkage. By iterating through these steps, the model describes the spatial-temporal behavior of the tumor, including cell density changes, development of hypoxic cores or tumor reoxygenation arising from changes in the vascular fraction as well as from changes in the oxygen consumption. Oxygenation within each voxel is described by the oxygen histograms calculated by TOM.

#### Virtual tumors

**Fig 1:**
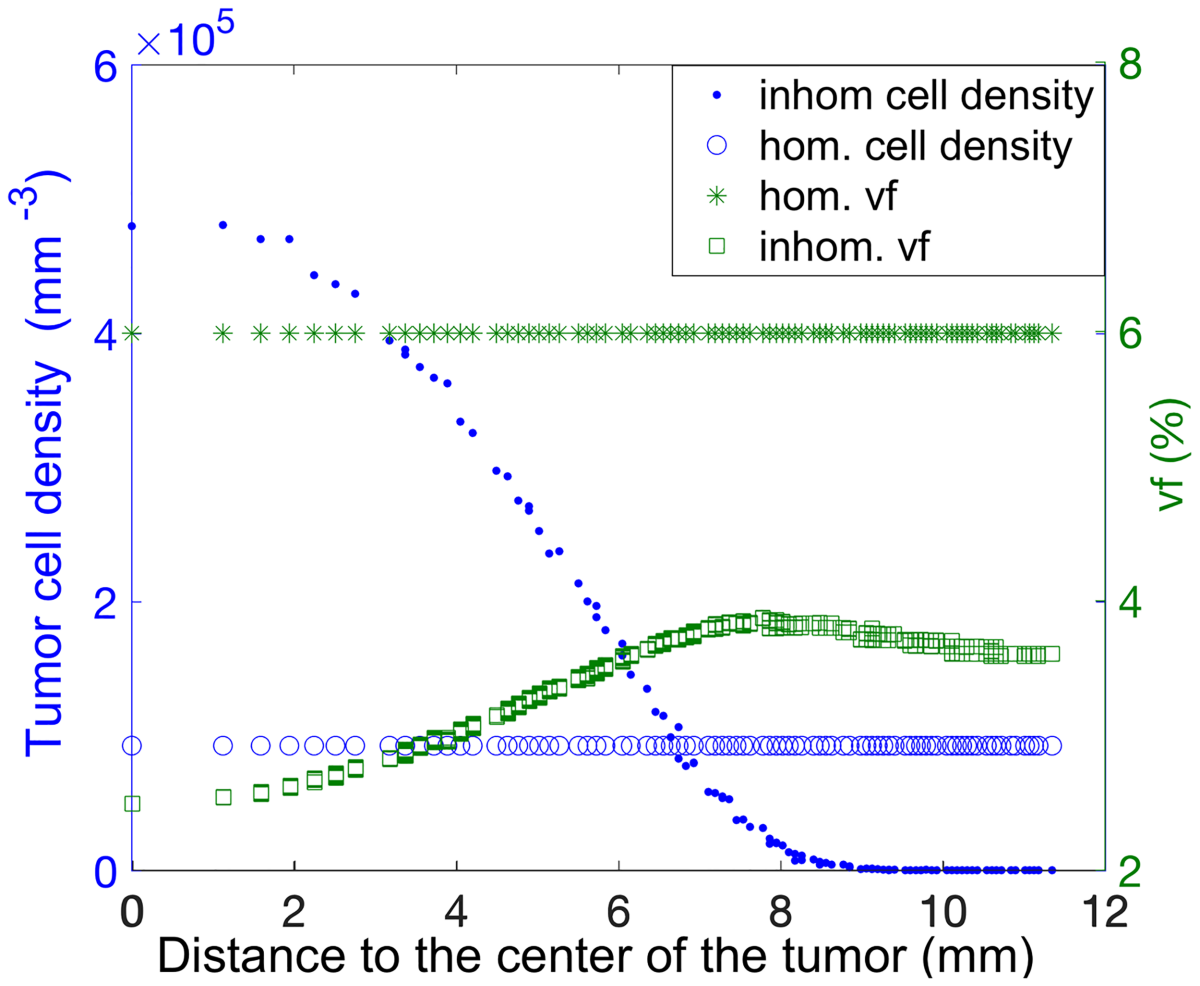
Radial distribution of vascular fraction and tumor cell density in the investigated virtual tumors prior to irradiation. Left axis: homogeneous (o) and inhomogeneous (.) tumor cell density distributions, *ρ*. Right axis: homogeneous (*) and inhomogeneous (□) vascular fraction distributions, vf. Each tumor consist of a combination of one vf and *ρ* profiles (see text for details).

Starting from a single cell, the TRM was used to grow a spherical virtual tumor of approximately 2 cm in diameter using parameters specific for a H&N-tumor. During this growth, the tumor develops a central hypoxic core of 1 cm diameter, corresponding to the gross tumor, and a 0.5 cm rim, with an increased oxygenation, representing the microscopic extension of the primary tumor. The tumor consists of 3888 cubic voxels (side length 1.124 mm) with an average cell density of *μ* = 10^6^ cells/mm^3^ [53]. The tumor cell density, *ρ*, decreases at the tumor border such that *ρ* ranges from 1 to approximately 5 × 10^5^ cells/mm^3^ (see Fig 1). The resting cells in the tumor voxels are capillary cells, according to the vf, and normal cells. This virtual tumor will be referred to as tumor 1 (T1).

To study the impact of the tumor heterogeneity (distribution of *ρ* and vf) on the outcome of DPBN treatments, results for the tumor T1 were compared with those achieved for other three additional virtual tumors, referred to as T2, T3 and T4. These tumors differed either in the distribution of *ρ* or vf, or in the distribution of both (see Fig 1). As a result, the following tumors were investigated: T1) non-uniform distribution of *ρ* and vf; T2) uniform distribution of *ρ* and vf; T3) uniform distritubion of *ρ* and non-uniform distribution of vf and T4) non-uniform distribution of *ρ* and uniform distribution of vf.

All tumors were defined to have the same number of tumor cells. The value of vf for the uniform distribution was selected to be 6%, which is a representative value for well oxygenated tumors [54, 55]. With this vf, the oxygen effect on the optimization process is expected to be negligible. The value of *ρ*, used for the uniform distribution in T2 and T3 was calculated from the total number of tumor cells in T1. The biological parameters values used for the simulation of the radiation response with the TRM were the same for all tumors.

#### Biological parameters in the TRM

For the simulation of tumor response, parameter values representative for H&N tumors were taken from the literature (Table 1). For this, a tumor cell doubling time (*t*_p_) of 1200 h was used at the beginning of the treatment. This slow tumor proliferation is in accordance with the Gompertzian growth of macroscopic tumors [56]. After two weeks of treatment, *t*_p_ was changed to 120 h to simulate radiation-induced accelerated repopulation [57]. The capillary cell doubling time (*t*_*a*_) was set to 612 hours to simulate the slow angiogenesis process [58, 59]. The rate of dead cells resorption, characterized by the half time *t*_r_, was considered to be equal to 168 h [60]. The radiosensitivity parameters of the LQ model were selected to be *α* = 0.35 Gy^−1^ and *β* = 0.035 Gy^−2^, respectively [61, 62]. Additionally, a *σ*_*α*_ equal to 0.05 Gy^−1^ was used to consider interpatient radiosensitivity variations [63, 64]. Finally, the parameter values associated to the OERs (Eq 2) were selected [49].

**Table 1:** Biological parameters used for the simulations.

Parameters	Symbol	Value
Average cell density	*μ*	10^6^ cells/mm^3^ [53]
Tumor cell proliferation doubling time	*t*_p_	120 and 1200 hours [56, 57]
Angiogenesis proliferation doubling time	*t*_a_	612 hours [58, 59]
Half time of dead cell resorption	*t*_r_	168 hours [60]
Radiosensitivity LQ-model parameters	*α*	0.35 Gy^−1^ [61, 62]
	*β*	0.035 Gy^−2^ [61, 62]
Normal std of the *α* parameter	*σ*_*α*_	0.05 Gy^−1^ [63, 64]
Maximum value of the OER for *α*	$OER_{\alpha_{m}}$	2.5 [49]
Maximum value of the OER for *β*	$OER_{\beta_{m}}$	3 [49]
OER slope parameter	*k*	3.28 mmHg [49]

Considering Eqs (3)–(5) and the employed tumor model, the dose optimization was carried out considering not only the oxygen-related variation of cell radiosensitivity in the tumor, but also the distribution of tumor cells density.

### Simulation studies

For each of the described virtual tumors (T1-T4), treatment response was calculated using a uniform dose distribution of 2 Gy per fraction as well as inhomogeneous dose distributions optimized using either OF_surv_ or OF_std_ (keeping the average dose equal to 2 Gy). A conventional fractionation scheme consisting of one daily fraction with two-day breaks on weekends, starting on a Monday, was simulated. These simulations were used to analyze the effect of the different objective functions in combination with different tumor characteristics.

**Fig 2:**
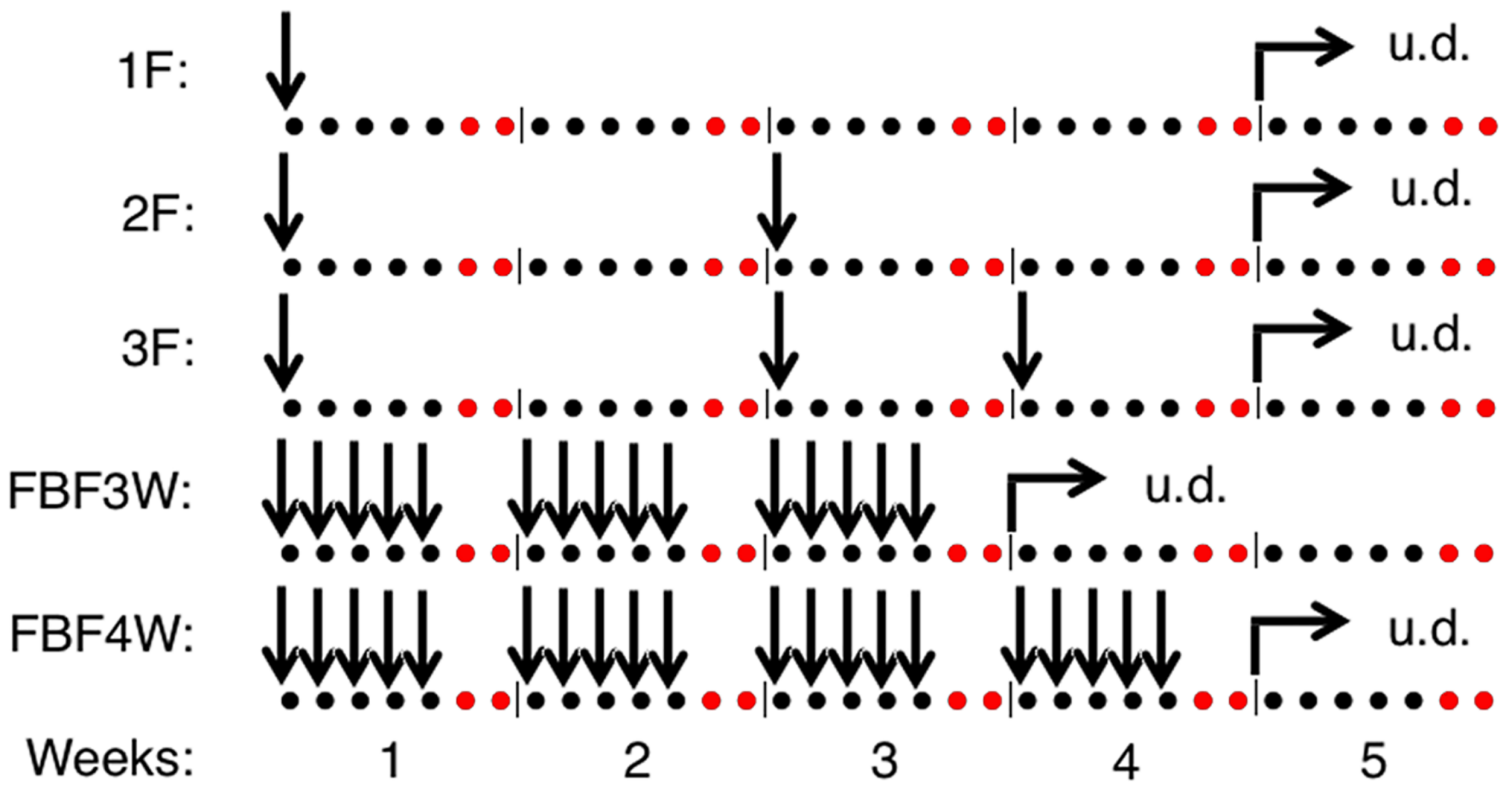
Time points of dose optimization within the investigated treatment schedules. Vertical arrows indicate the days (represented with dots) when dose optimization was performed. The optimized distributions are applied in all the treatment fractions from the day of optimization until a new optimization is performed. Note: No irradiations were performed at weekend (dots in red) and after 3 weeks (FBF3W) or 4 weeks (all other schedules), fractions were delivered with uniform dose (u.d. = 2Gy). The response of each virtual tumor was simulated for a uniform dose distribution as well as for the five adaptive schemes (1F, 2F, 3F, FBF3W and FBF4W) optimizing the dose distribution either with OF_surv_ and OF_std_.

In general, the dose distribution resulting from the optimization will depend on the biological status of the tumor, which will change during the course of irradiation. In terms of biologically adapted radiotherapy, these changes could be taken into account by daily optimization. For clinical implementation, however, this would require a daily assessment of the biological status of the tumor, *e.g.* by imaging. As this may not be feasible and since the changes in the tumor may not be so fast, a more practical approach would be to optimize the dose distribution only at certain time points of the treatment schedule. Although the best time for replanning is still a matter of debate for the clinical implementation of dose painting, there is some consensus towards doing it 1-3 weeks after treatment start [19]. We therefore simulated different adaptive schemes (see Fig 2): 1F) One fraction optimization: the dose distribution is optimized only once, at the beginning of the treatment; 2F) Two fractions optimization: the optimization is done at the beginning of the treatment and after 2 weeks of treatment; 3F) Three fractions optimization: the optimization is done at the beginning of the treatment, after 2 weeks of treatment and after 3 weeks of treatment; FBF3W) Fraction by fraction optimization: the optimization is done for every single fraction within the first 3 weeks and FBF4W) Fraction by fraction optimization: the optimization is done for every single fraction within the first 4 weeks. Uniform 2 Gy dose distributions were delivered after the 4th week of treatment in all cases with the exception of the FBF3W scheme, that continued treatment with uniform distributions already after the 3rd week.

#### Treatment outcome evaluation and statistical analysis

The response of the tumors was quantified in terms of cell survival and TCP curves. For the TCP calculations, tumor response was simulated at different dose levels for a population of 30 tumors per dose level using a varying intrinsic radiosensitivity described by the parameter *σ*_*α*_ in Table 1 [63, 64]. The dose level increment was realized by increasing the number of fractions rather than the fractional dose. The DPBN dose distributions were calculated by running the TRM with the mean radiosensitivity alpha parameter. An individual tumor was considered as controlled if no tumor cell survived. For each dose level, control rates were calculated as the ratio of controlled to total number of irradiated tumors. TCP curves were fitted to the control rates using a univariate logistic regression model, based on the mean dose to the tumor for each patient. TCP curves were characterized by tumor control dose, D50 (dose at 50% TCP), which can be derived as the ratio of the two fitting coefficients of the logistic model [65]. Regarding the statistical analysis, a bootstrapping method was used to estimate the uncertainty associated to the D50 calculations [66]. Uncertainties are expressed with two standard deviations (coverage factor, k = 2). For each tumor type and DPBN adaptive scheme, treatment gain was calculated as the difference of D50 between the conventional uniform dose and the DPBN treatment (this is, *gain* = *D*50_conv_ − *D*50_DPBN_).

## Results

### Tumors response to uniform dose distributions

**Fig 3:**
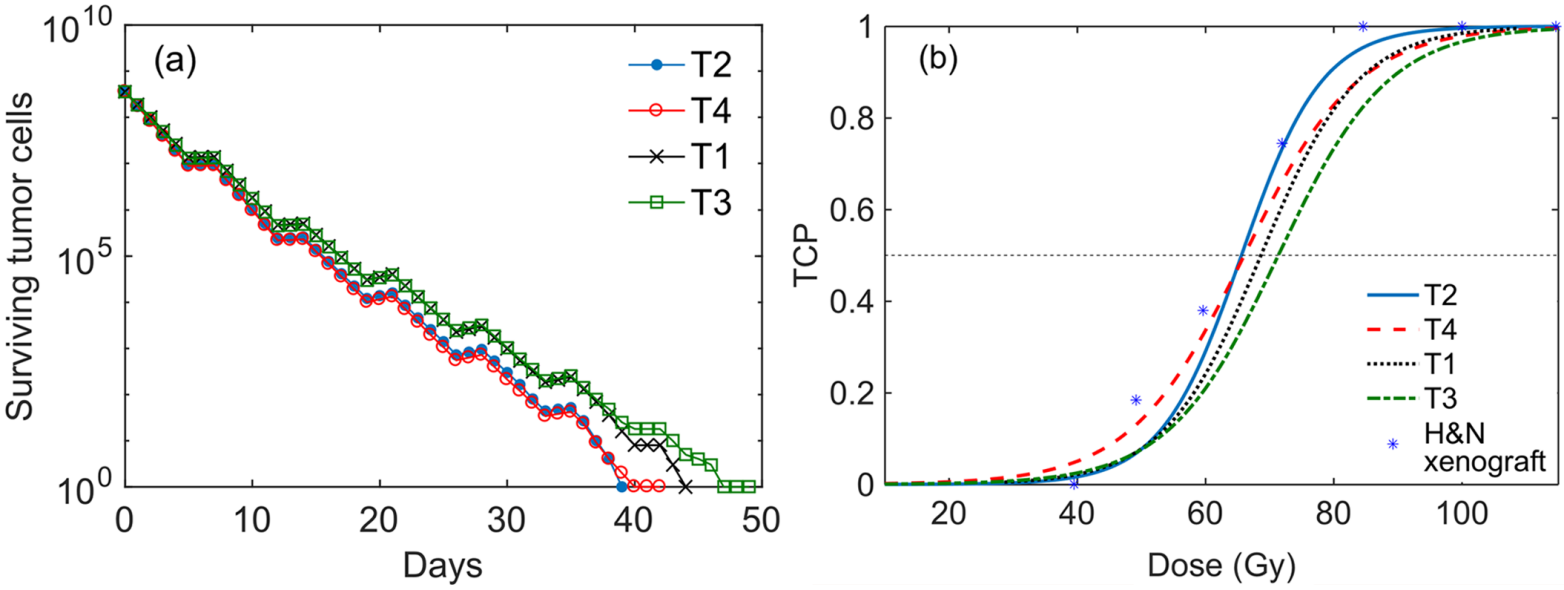
Tumor response to a uniform dose distribution. (a) Number of surviving tumor cells with time for the 4 tumor types irradiated with a uniform dose distribution. The weekend treatment breaks lead to small plateaus in the cells survival curves, in which the number of tumor cells increases slightly due to proliferation. (b) TCP curves for the simulated tumors and experimental response of a 200 *mm*^3^ xenograft of the H&N FaDu line [67]. The D50_conv_ values (in Gy) of the simulated TCP curves are 68.6 ± 1.4, 65.6 ± 2.1, 71.3 ± 2.0 and 66.0 ± 2.0, for T1, T2, T3 and T4 respectively.

The simulation of the response to conventional treatments with uniform dose distributions leads only to slightly different responses for the different tumors (see Fig 3A). As all tumors have the same number of tumor cells, a difference is only seen between the well oxygenated tumors (T2, T4) and the tumors with a hypoxic core (T1, T3). In accordance with this, the well oxygenated tumors (T2 and T4) have the same D50 values, which are lower than the D50 values of the hypoxic tumors, see Fig 3B. Focusing in the hypoxic tumors (T1 and T3), the slightly better outcome observed for T1 with respect to T3 is due to the observed higher degree of reoxygenation occurring in this tumor during the treatment. These TCP curves are in accordance with those observed from preclinical studies involving H&N xenografts from an intermediate radiosensitivity cell line (FaDu) and with a volume similar to the virtual tumors considered in our work [67]. The radiosensitivity *α* and *β* parameter values used in our simulations were taken from the literature [61, 62] and not specifically modified to fit to this experimental curve.

**Fig 4:**
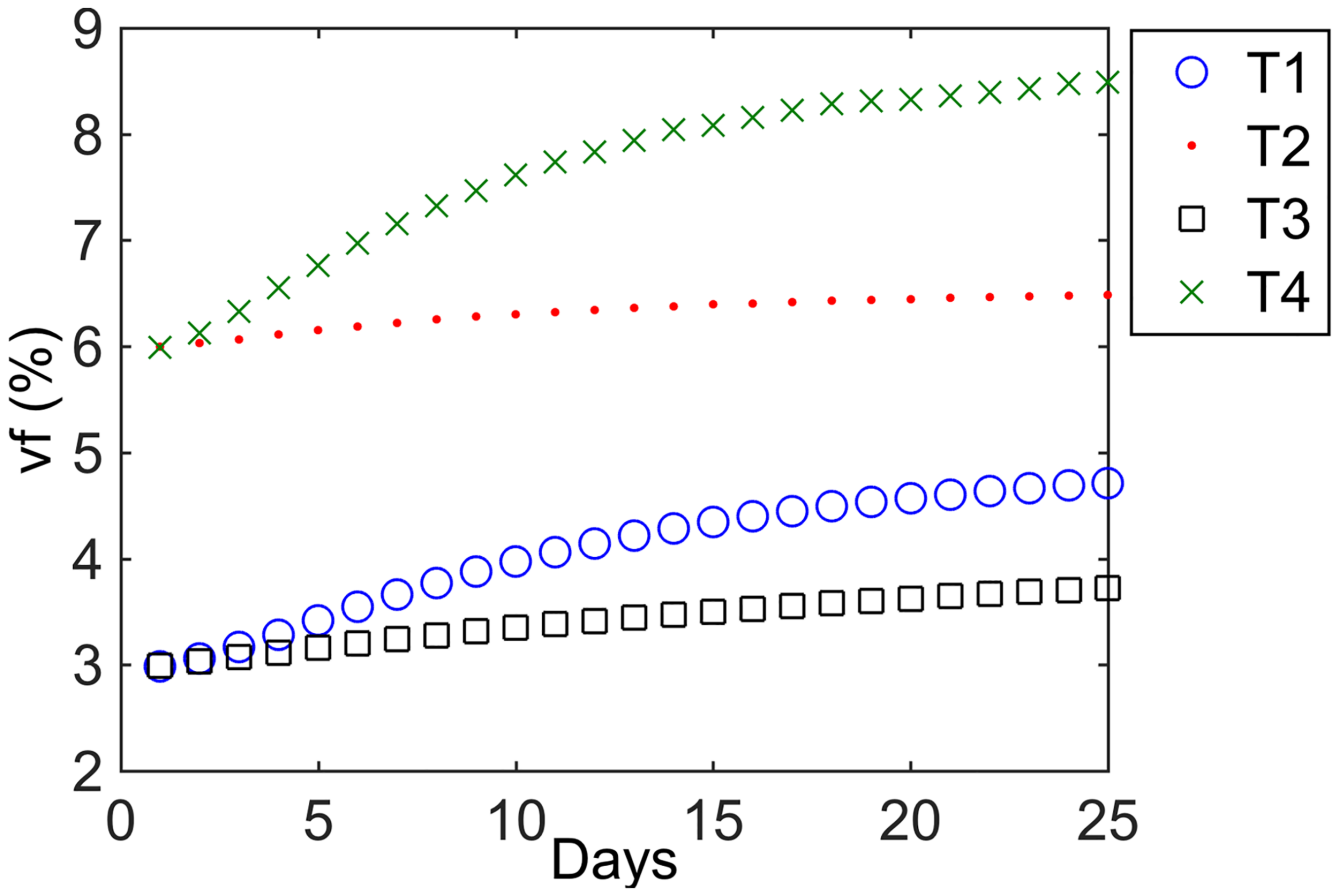
Radiation induced reoxygenation in the investigated virtual tumors. Evolution of the vascular fraction (averaged in the 1 cm diameter tumor core) for the 4 tumors when irradiated with a uniform dose distribution.

To illustrate the evolution of oxygenation during the course of the treatments, the status of the tumor core vf during the treatment is shown in Fig 4. The higher reoxygenation capability observed for the tumors with non-uniform tumor cell density (T1 and T4 reoxygenating more than T3 and T2, respectively) is due to the larger number of tumor cells killed in regions with high tumor cell density, which leads to a more pronounced tumor shrinkage associated with a stronger reoxygenation. This relationship has not been clinically proven and might not represent the true response of clinical tumors. However, as the reoxygenation-related radiosensitization might affect the treatment gain associated to DPBN, the use of virtual tumors with these distinct characteristics is considered of interest for the purpose of this work.

### Tumors response to DPBN treatments

**Fig 5:**
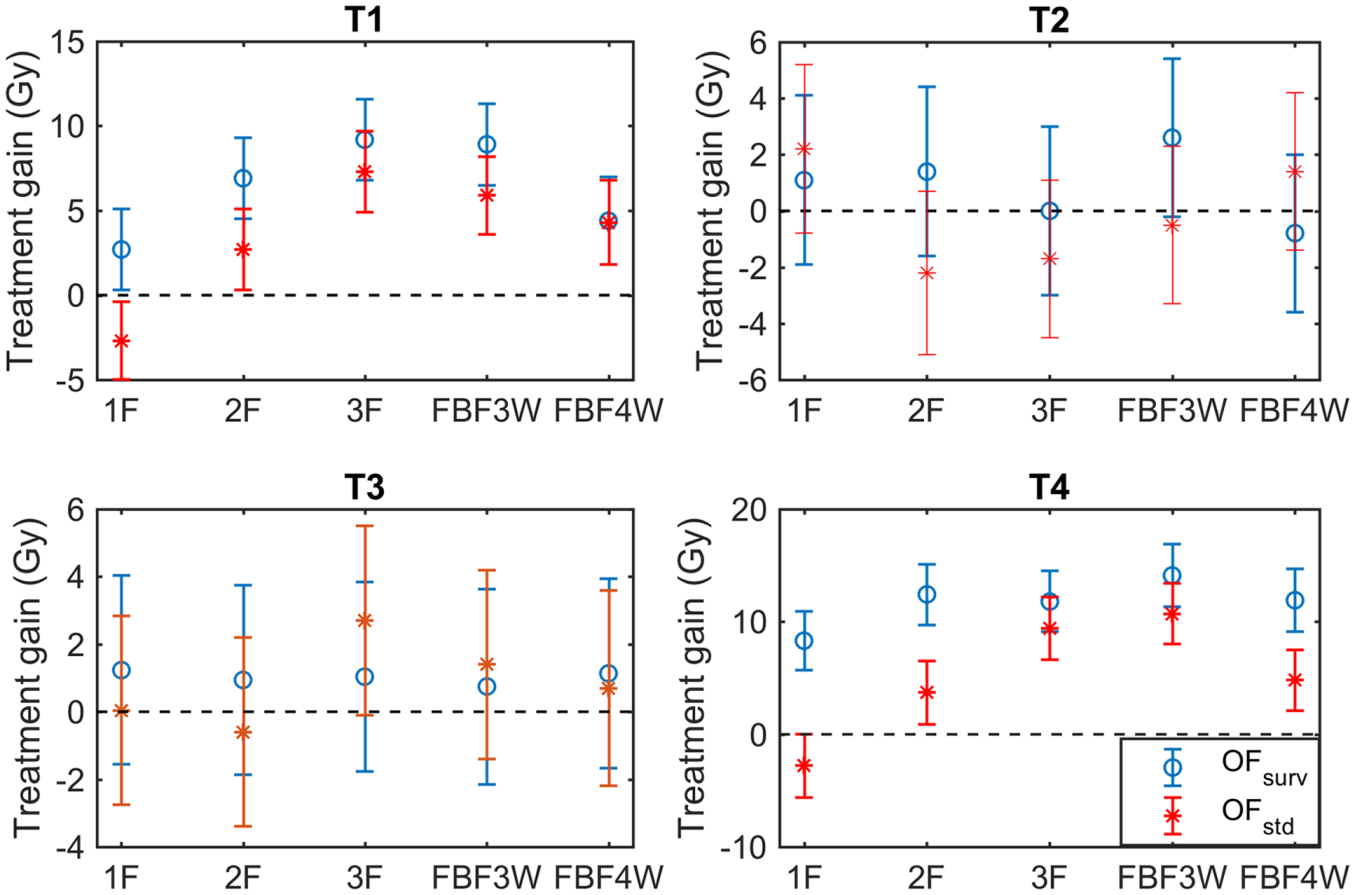
Treatment gains. Treatment gains obtained for the studied tumors (T1-T4) using dose distributions optimized with either OF_surv_ (∘) or OF_std_ (*) under different adaptive schemes.

The response of the tumors to the DPBN treatments is presented in Fig 5 in terms treatment gain (T1 and T4, of higher reoxygenation capability, and T2 and T3, of lower reoxygenation capability). It is noted that DPBN applied to T1 and T4 exhibits significantly larger treatment gains, than for T2 and T3, where treatment gains are comparable with zero within uncertainties. Detailed D50 values leading to these treatment gains can be found in S1 Table.

#### Effect of the optimization objective function

Fig 5 shows that treatment gains are systematically larger for the DPBN treatments using OF_surv_ (minimization of survival) than for OF_std_ (minimization of heterogeneity). This is true under all the adaptive schemes investigated for tumors T1 and T4. Although treatment gains of T2 and T3 are not significant, they also exhibit systematically higher values with OF_surv_ in most of the cases.

#### Effect of different adaptive schemes

The responses observed under the different adaptive schemes depend again on the tumor type. For T2 and T3, outcomes for the conventional and the DPBN treatments are very similar (treatment gains comparable with zero within uncertainties, see Fig 5). Further analysis revealed that this observation is a result of the degree of vf heterogeneity and not due to the limited variation of dose allowed in this study (25% of 2 Gy). On the other hand, the DPBN treatments delivered to T1 and T4 exhibit treatment gains which generally increased with the number of dose distribution optimizations performed during the treatment (*gain*_1*F*_ < *gain*_2*F*_ < *gain*_3*F*_ < *gain*_*FBF*3*W*_). However, only small differences between 3F and FBF3W were observed, and treatment gains for FBF4W decreased again relative to FBF3W. This is observed for both objective functions and was found to be related to the following reasons: i) As the DPBN treatment was optimized assuming a population-averaged radiosensitivity, the resulting dose distribution is slightly suboptimal for the individual tumors used to estimate TCP, ii) Another factor contributing to this is the small differences arising from the stochasticity of the cell killing, which becomes important towards the end of the treatment (when the number of tumor cells is small). For this reason, dose distributions were only optimized up to 4 weeks after the start of treatment. This effect also contributes to the finding that the treatment gains for FBF4W are not signicantly larger than for FBF3W.

**Fig 6:**
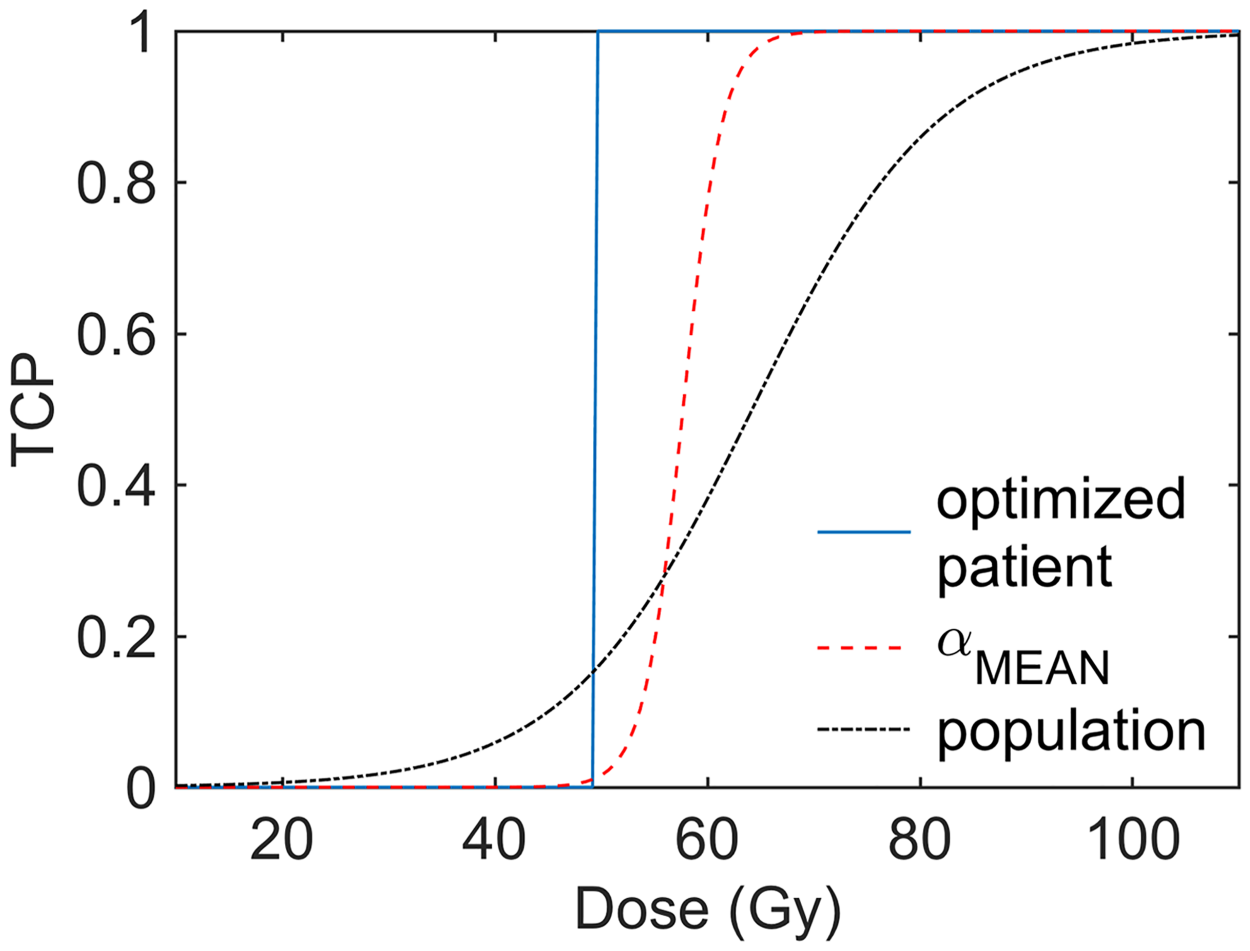
Sthocastic and population radiosensitivity variability effects. Response curves for tumor T1 when irradiated with the FBF4W scheme using OF_surv_. Solid line, treatment response achieved when the treatment is optimized using the population-averaged radiosensitivity *α*; dashed line, TCP curve calculated using the same population-averaged radiosensitivity (the cell killing stochasticity produces the shallowing and displacement towards higher dose of the response curve); and dashed-dotted line, TCP curve calculated for a population with a normally distributed *α* radiosensitivity parameter (further shallowing and displacement).

To illustrate this effect, Fig 6 shows the response curves of T1 when irradiated with the FBF4W adaptive scheme using OF_surv_ under different assumptions. (i) When the dose distributions are optimized using one single tumor having the population-averaged radiosensitivity *α*, the tumor is controlled with 50 Gy. (ii) If the same treatment is delivered repeated times to this tumor to build a TCP curve, the cell killing stochasticity makes the response curve to become slightly shallow and D50 is (57.7 ± 1.0) Gy. (iii) If the radiosensitivity is additionally assumed to be normally distributed within a tumor population, D50 increases further to (64.2 ± 2.2) Gy and the slope is even more shallow.

#### Effect of tumor inhomogeneities

**Fig 7:**
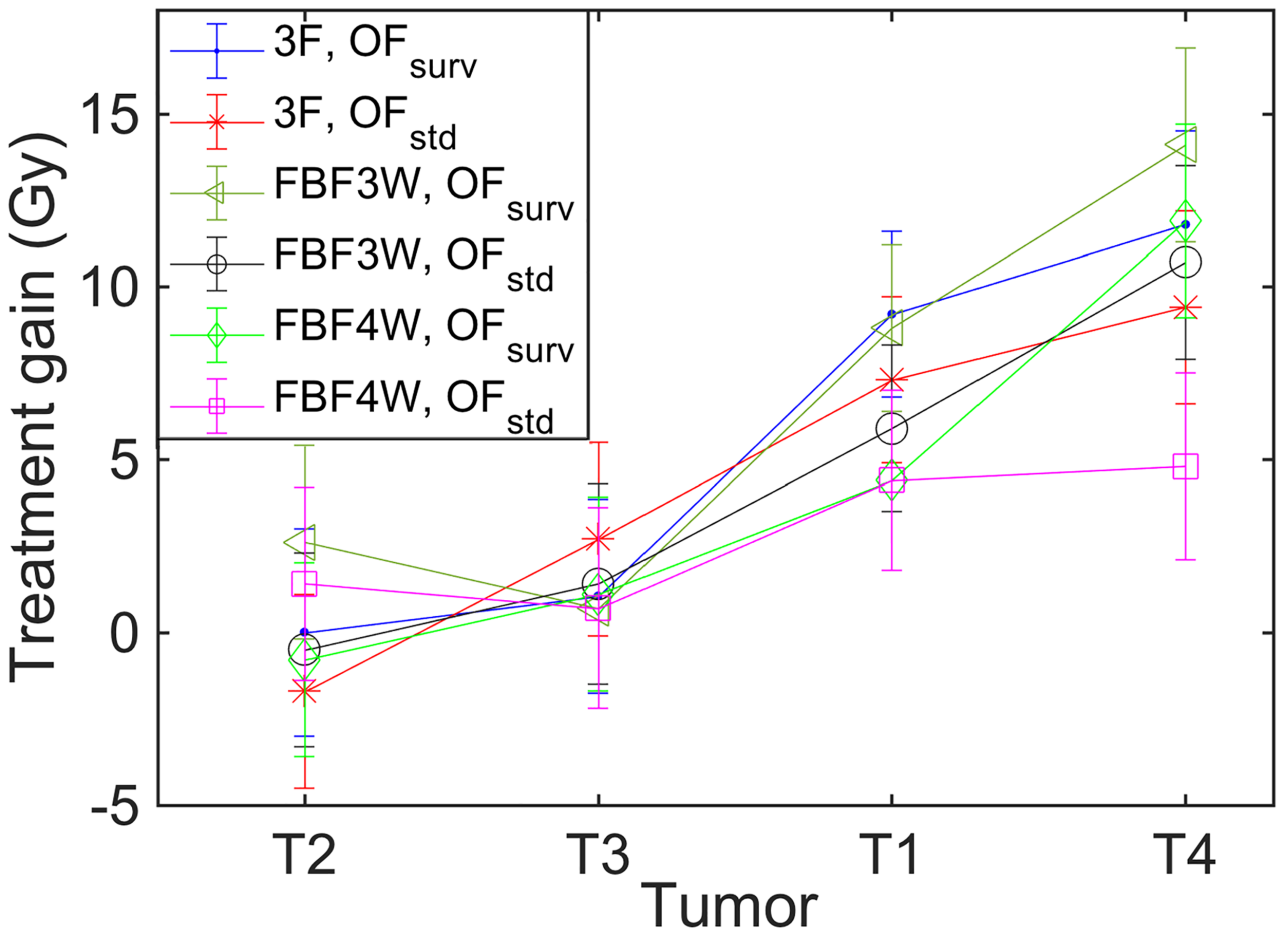
Impact of the adaptive scheme on treatment gain. Treatment gains associated to the DPBN treatments under the adaptive schemes 3F, FBF3W and FBF4W. Tumors ordered on the x-axis by ascending reoxygenation capability.

As shown above, the treatment gains achieved with the DPBN treatments were different for the 4 tumors, with values comparable with zero in some cases (T2 and T3). When the results for the 4 tumors are compared, a relationship between tumor reoxygenation capability and treatment gain can be observed: the higher the reoxygenation capability, the higher the treatment gain obtained from the DPBN treatment. Fig 7 shows the treatment gains associated to the DPBN treatments for both OFs and for the adaptive schemes leading to the higher treatment gains (3F, FBF3W and FBF4W). The tumors are ranked on the x-axis according to their reoxygenation capability (reox_*T*2_ < reox_*T*3_ < reox_*T*1_ < reox_*T*4_).

## Discussion

### Comparison with other studies and limitations of this work

The present work studies DPBN by using a in-silico tumor response model that simultaneously considers several biological processes in the tumor during treatment. These processes are: oxygen-dependent cell survival after irradiation, proliferation of tumor cells, hypoxia-induced angiogenesis, resorption of dead cells, and tumor growth or shrinkage. These effects show complex interaction with tumor reoxygenation [46]. To our knowledge, no other study on the modelling of dose painting treatments considered these interacting biological processes altogether. Previous works either did not consider reoxygenation [13, 20, 22, 34], or simulated it in a simplified way [23, 24, 47]. For example, some of them neglected tumor proliferation during treatment [24, 47] or decoupled it from reoxygenation [23], although both processes are known to be linked [68].

In this work we show that the dynamics of the biological processes during irradiation strongly affect the treatment results. Thus, to accurately model the radiation response of clinical tumors treated with radiotherapy using dose painting, three main aspects must be considered: i) a proper representation of the tumor oxygenation at a subvoxel scale [24, 49], ii) the spatial distribution of tumor cells [9, 20], and iii) realistic values of the biological parameters. In addition to the above mentioned aspects, we showed that the choice of the OF used for the DPBN optimization is also important. Minimizing the survival [9, 20, 24] was observed to lead to better treatment outcomes than minimizing the spatial heterogeneity of the surviving tumor cells, which had been investigated by [23, 37, 47]. The reason for this is that dose distributions calculated using OF_std_ change more drastically from fraction to fraction than those calculated using OF_surv_ (data not shown). Thus, when the tumor characteristics are measured only once or twice during treatment, the solution from OF_surv_ is closer to the optimal distribution that would have been calculated for the intermediate fractions. In this situation (only one or two optimizations), the use of OF_std_ would lead to lower local tumor control rates than the use of a uniform dose distribution. Consequently, the use of OF_surv_ would present a better choice for DPBN treatments.

In the study of different adaptive schemes, better treatment outcomes are observed when optimizations are performed at several time points of the treatment schedule, which is in agreement with the work of Sovik *et al.* 2007 [23]. The difference between fraction by fraction optimization schemes and optimizing 3 times (at the beginning of weeks 1, 3 and 4), was however found to be small. This is of great clinical advantage as a 3F scenario would be much implementable in the clinic, while performing daily measurements of the tumor characteristics with subsequent optimization is unfeasible. Other factors discouraging the use of fraction by fraction adapted dose distributions would be: i) uncertainties associated the measurement of tumor characteristics with molecular imaging (with radiation induced signal due to inflammation affecting some tenchiques like FDG) ii) lack of knowledge of individual tumor radiosensitivities, and iii) cell killing stochasticity, which may affect treatment response (being not reasonable to perform DPBN) in the late phase of the treatment.

Unfortunately, comparison of our results with experimental data is currently not possible. Several clinical trials have already implemented similar promising (2F or 3F) dose redistribution adaptive schemes, but for some of them, like the ARTFORCE trial [39], outcome data is still incomplete. Some other trials aimed at reducing normal tissue toxicity and thus results from these works are not comparable with ours [28, 45]. Amongst the few publications quantifying dose painting related treatment gain, the work recently published by Kong *et al.* shows that one (mid-treatment) FDG-PET based adaptation can result in locoregional tumor control improvement for patients with locally advanced Non-Small-Cell Lung Cancer [40]. However, this trial involved dose escalation and comparison of results with our dose redistribution approach is neither possible. One preclinical study [69] has investigated the use of dose redistribution with larger heterogeneities than the 25% allowed in our work. Trani *et al.* applied DPBC using 40% and 60% dose heterogeneity constraints, based on a pretreatment FDG-PET image, for single fraction irradiation of rhabdomyosarcomas [69]. Such dose redistributions were observed to be detrimental for tumor control with respect to the use of homogeneous dose distributions. This work did not involved the use of any radiobiology based dose distribution optimization though. Other trials have been focused on dose escalation, frequently aiming at endpoints different to tumor control [25, 41, 43, 44, 70].

This work was developed for the DPBN technique because it allows a more general voxel-based heterogeneity analysis. Results for the DPBC technique, also appropriate for tumors with relatively large subvolumes of uniform characteristics [71], should not be significantly different. Treatment gain values may change, but the authors would expect that DPBC plans using several (few) dose distribution optimizations should still be more beneficial for tumors with a higher reoxygenation capability than for poorly reoxygenating tumors. The effect of the optimization objective functions should also be the same for DPBN and DPBC.

We should notice that, in spite of the variety of interplaying dynamic biological processes considered in our work, the biological mechanisms arising in tumors during radiotherapy are very complex and not yet well understood, and our model makes simplifications about tumor growth and response. For example, only one type of tumor cells is considered, but it is well known that tumor cells with different degrees of differentiation conform the tumor, and that the initially small subpopulation of tumor stem cells plays an important role both in response and tumor repopulation [72]. We simulate, however, an average tumor cell radiosensitivity and proliferation parameter values based on experimental doubling times. Moreover, accelerated repopulation was modelled by using a single kick-off and doubling time, but there is evidence that such parameters may depend on tumor stage, number of viable tumor stem cells or hypoxia status [57, 73]. Tumor shrinkage in our model is due to undirected cell exchange, which may be also an oversimplification of the underlying process [74]. Cell death kinetics can affect the reoxygenation rate of tumors, but this effect is not relevant for conventional fractionation and vf above 3% [75].

A relevant aspect in DPBN is the choice of optimization constraints. The results presented here were derived from optimizations subjected to two constraints: i) a constant average dose constraint (2 Gy/fraction) and ii) a dose heterogeneity constraint of ±25% of 2 Gy, allowing doses from 1.5 to 2.5 Gy in each tumor voxel. No limits were however established for the allowed dose differences between neighboring voxels. Modern radiotherapy techniques are able to deliver strongly modulated dose distributions, however, the spatial resolution in the order of 5 mm may still be somewhat below that of the voxel size used in the TRM. Therefore, the use of smoother dose distributions might be clinically more feasible. Despite these discussed limitations, the trends observed in this work should not be qualitatively different from those observed in a clinical scenario.

## Conclusion

Our study of DPBN with a computer-based tumor response model allowed us to gain insight into some factors affecting the treatment gain, like the optimization objective function, the tumor reoxygenation capability and the implemented adaptive scheme. Our study shows that tumors with high reoxygenation capability benefit more from DPBN. Additionally, the treatment gains of DPBN treatments in which dose distributions are optimized once a week are similar to those achieved with daily optimization. This indicates that only a few weekly optimizations, which is clinically more feasible, may be sufficient to improve the response of hypoxic tumors. This work shows that the dynamics of the biological processes arising in tumors during treatment have a relevant effect on dose distribution optimization. This evidences the need of not only a proper understanding of these processes but also quantitative information from functional imaging or any other methods.

Regarding the objective function, dose distributions optimized minimizing survival lead to better treatment outcomes than those optimized minimizing the spatial heterogeneity of the tumor cell survival.

## Supporting information

S1 Table Treatment outcomes data. Treatment outcomes for the investigated tumors quantified in terms of D50 values and treatment gains of DPBN treatments relative to irradiation with uniform dose. All values are expressed in Gy. DPBN treatments were optimized either with OF_surv_ or OF_std_.
(PDF)
