# Supplementary material for: Impact of different biologically-adapted radiotherapy strategies on tumor control evaluated with a tumor response model
Source: PLoS One. 2018 Apr 26;13(4):e0196310. doi: 10.1371/journal.pone.0196310 (PMC5919644; doi:10.1371/journal.pone.0196310)
Supplement: S1 Table — Treatment outcomes for the investigated tumors quantified in terms of D50 values and treatment gains of DPBN treatments relative to irradiation with uniform dose. All values are expressed in Gy. DPBN treatments were optimized either with OFsurv or OFstd. (PDF) [file pone.0196310.s001.pdf]

S1A Table. Treatment outcomes of tumors with high reoxygenation capability. Treatment outcomes for T1 and T4, quantified in terms of D50 values and treatment gains of DPBN treatments relative to irradiation with uniform dose. All values are expressed in Gy. DPBN treatments were optimized either with OF_{surv} or OF_{std}.

		T1		T4	
	Adaptive scheme	D50	Gain	D50	Gain
OF_{surv}	1F	65.9 ± 1.9	2.7 ± 2.4	57.7 ± 1.7	8.3 ± 2.6
	2F	61.7 ± 1.9	6.9 ± 2.4	53.6 ± 1.8	12.4 ± 2.7
	3F	59.4 ± 1.9	9.2 ± 2.4	54.2 ± 1.8	11.8 ± 2.7
	FBF3W	59.8 ± 1.9	8.8 ± 2.4	51.9 ± 2.0	14.1 ± 2.7
	FBF4W	64.2 ± 2.2	4.4 ± 2.6	54.1 ± 1.9	11.9 ± 2.8
OF_{std}	1F	71.3 ± 1.8	-2.7 ± 2.3	68.8 ± 1.9	-2.8 ± 2.8
	2F	65.9 ± 1.9	2.7 ± 2.4	62.3 ± 2.0	3.7 ± 2.8
	3F	61.3 ± 1.9	7.3 ± 2.4	56.6 ± 2.0	9.4 ± 2.8
	FBF3W	62.7 ± 1.8	5.9 ± 2.3	55.3 ± 1.8	10.7 ± 2.7
	FBF4W	64.2 ± 2.1	4.4 ± 2.5	61.2 ± 1.8	4.8 ± 2.7
	Uniform dose	68.6 ± 1.4	–	66.0 ± 2.0	–

S1B Table. Treatment outcomes of tumors with low reoxygenation capability. Treatment outcomes for T2 and T3, quantified in terms of D50 values and treatment gains of DPBN treatments relative to irradiation with uniform dose. All values are expressed in Gy. DPBN treatments were optimized either with OF_{surv} or OF_{std}.

		T2		T3	
	Adaptive scheme	D50	Gain	D50	Gain
OF_{surv}	1F	64.5 ± 2.0	1.1 ± 2.9	70.1 ± 1.9	1.2 ± 2.8
	2F	64.2 ± 2.0	1.4 ± 2.9	70.4 ± 2.0	0.9 ± 2.8
	3F	65.6 ± 2.1	0.0 ± 3.0	70.3 ± 2.0	1.0 ± 2.8
	FBF3W	63.0 ± 1.9	2.6 ± 2.8	70.6 ± 1.9	0.7 ± 2.8
	FBF4W	66.4 ± 1.8	-0.8 ± 2.8	70.2 ± 2.1	1.1 ± 2.8
OF_{std}	1F	63.4 ± 2.1	2.2 ± 3.0	71.3 ± 2.0	0.0 ± 2.8
	2F	67.8 ± 2.0	-2.2 ± 2.9	71.9 ± 1.9	-0.6 ± 2.8
	3F	67.3 ± 1.9	-1.7 ± 2.8	68.6 ± 2.0	2.7 ± 2.8
	FBF3W	66.1 ± 1.9	-0.5 ± 2.8	69.9 ± 2.1	1.4 ± 2.9
	FBF4W	64.2 ± 1.9	1.4 ± 2.8	70.6 ± 1.9	0.7 ± 2.8
	Uniform dose	65.6 ± 2.1	–	71.3 ± 2.0	–
